# Supplementary figures and images for: Caffeic acid phenethyl ester protects Clostridioides difficile infection by toxin inhibition and microbiota modulation
Source: eLife. 2025 Jun 11;13:RP101757. doi: 10.7554/eLife.101757 (PMC12158428; doi:10.7554/eLife.101757)

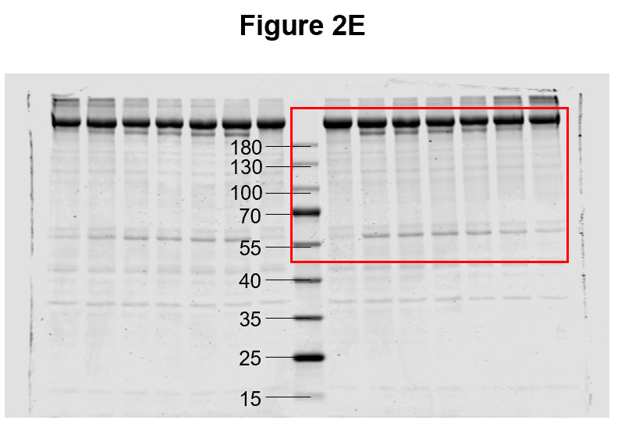

Supplement: Figure 2—source data 1. [file elife-101757-fig2-data1.zip › PDF file containing original western blots for Figure 2, indicating the relevant bands and treatments/Figure 2-source date 1.tif]

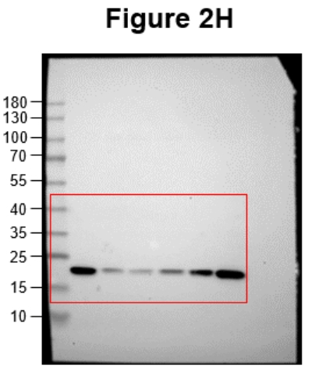

Supplement: Figure 2—source data 1. [file elife-101757-fig2-data1.zip › PDF file containing original western blots for Figure 2, indicating the relevant bands and treatments/Figure 2-source date 2.tif]

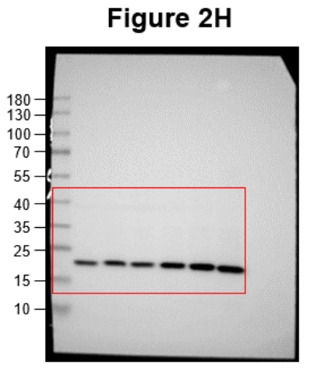

Supplement: Figure 2—source data 1. [file elife-101757-fig2-data1.zip › PDF file containing original western blots for Figure 2, indicating the relevant bands and treatments/Figure 2-source date 3.tif]

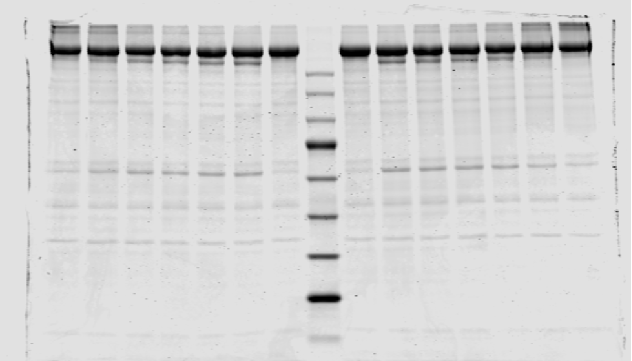

Supplement: Figure 2—source data 2. [file elife-101757-fig2-data2.zip › Original files for western blot analysis displayed in Figure 2/source data 1.tif]

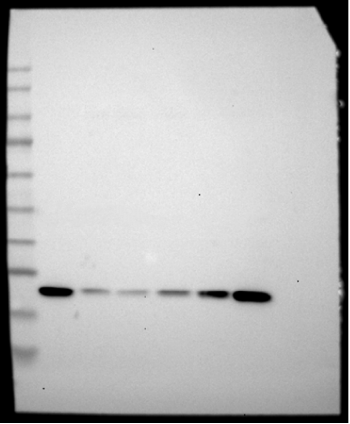

Supplement: Figure 2—source data 2. [file elife-101757-fig2-data2.zip › Original files for western blot analysis displayed in Figure 2/source data 2.tif]

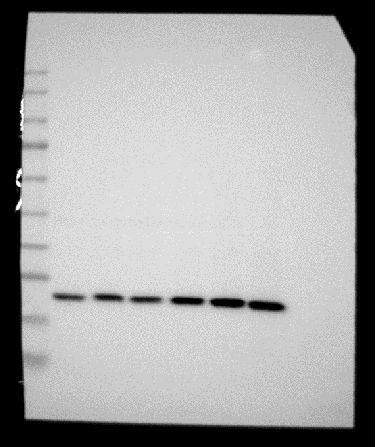

Supplement: Figure 2—source data 2. [file elife-101757-fig2-data2.zip › Original files for western blot analysis displayed in Figure 2/source data 3.tif]
