# Supplementary material for: Caffeic acid phenethyl ester protects Clostridioides difficile infection by toxin inhibition and microbiota modulation
Source: eLife. 2025 Jun 11;13:RP101757. doi: 10.7554/eLife.101757 (PMC12158428; doi:10.7554/eLife.101757)
Supplement: Supplementary file 1. [file elife-101757-supp1.docx]

**Supplementary File 1A.** **IC_50_ of the caffeic acid and its derivate on TcdB-mediated cell-rounding.**

| **Compound** | **Structure** | **IC_50_ (μg/mL)** |
| --- | --- | --- |
| Caffeic acid | 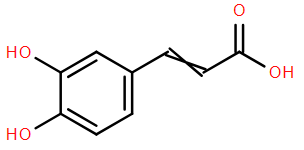 | 527.5 |
| Ethyl caffeate | 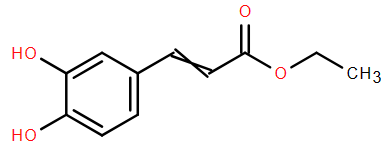 | 5.9 |
| CAPE | 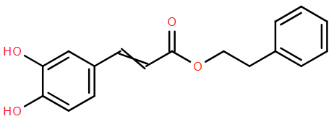 | 3.0 |
| Echinacoside | 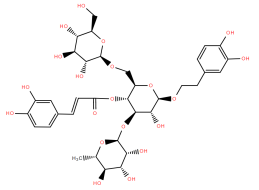 | 5.1 |
| Rosmarinic acid | 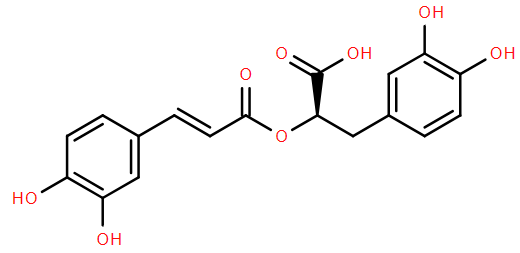 | 198.1 |
| Salvianolic acid | 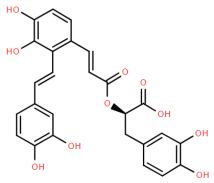 | 3.4 |
| Verbascoside | 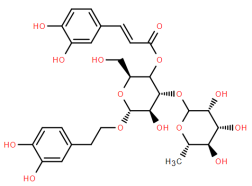 | 5.4 |

**Supplementary File 1B. Primers used in this study.**

| **Primers** | **Sequences** | **Notes** | |
| --- | --- | --- | --- |
| YG101 | ctgGGATCCATGAGTTTAGTTAATAG | *gtd* BamHI-F | |
| YG102 | ctgGTCGACCTAAAGAGAACCTTCAA | | *gtd* SalI-R |
| YG103 | CAGAAGCAGCAGCTGCATTCCACCTTTCTACCAACTCTTGTTC | | *gtd*_L265A_ -F |
| YG104 | GAACAAGAGTTGGTAGAAAGGTGGAATGCAGCTGCTGCTTCTG | | *gtd*_L265A_ -R |
| YG105 | GTATTCCTGGTAACATATCAACAGCTAAATACATACCACCAATTTCT | | *gtd*_D286A_ -F |
| YG106 | AGAAATTGGTGGTATGTATTTAGCTGTTGATATGTTACCAGGAATAC | | *gtd*_D286A_ -R |
| YG107 | CAGGGCCACTTAAGTTAATAGCAGTTTTAACATCTGGGAAGAA | | *gtd*_T465A_-F |
| YG108 | TTCTTCCCAGATGTTAAAACTGCTATTAACTTAAGTGGCCCTG | | *gtd*_T465A_-R |
| YG109 | TGCTTCAGGGCCACTTAAGTTAGCAGTAGTTTTAACATCTGGGAAG | | *gtd*_I466A_-F |
| YG110 | CTTCCCAGATGTTAAAACTACTGCTAACTTAAGTGGCCCTGAAGCA | | *gtd*_I466A_-R |
| YG111 | CTTGCATCGTCAAATGACCATGCGCTAGCCATTTCTTGTTCAGT | | *gtd*_L519_ -F |
| YG112 | ACTGAACAAGAAATGGCTAGCGCATGGTCATTTGACGATGCAAG | | *gtd*_L519A_ -R |
| YG113 | CTCTTGCATCGTCAAATGACGCTAAGCTAGCCATTTCTTGTTC | | *gtd*_W520A_ -F |
| YG114 | GAACAAGAAATGGCTAGCTTAGCGTCATTTGACGATGCAAGAG | | *gtd*_W520A_-R |

**Supplementary File 1C. Sequence of primers used for Reverse transcriptase PCR (RT-PCR) analysis.**

| **Gene** | **Primer** | **Sequence (5’-3’)** |
| --- | --- | --- |
| *tcdB* | Forward | TAGCAAGAAGTTCAGAGAGAGGAT |
|  | Reverse | CTTGTATTTCACCATCTCCAGGA |
| *16S* | Forward | GCGTAGGCGGTCTTTCAAG |
|  | Reverse | TCGCAACTGGTGTTCCTCC |
